# Activation of the hypothalamic-pituitary-adrenal stress axis induces cellular oxidative stress

**DOI:** 10.3389/fnins.2014.00456

**Published:** 2015-01-19

**Authors:** Jereme G. Spiers, Hsiao-Jou Cortina Chen, Conrad Sernia, Nickolas A. Lavidis

**Affiliations:** School of Biomedical Sciences, The University of QueenslandBrisbane, QLD, Australia

**Keywords:** corticosterone, hypothalamic-pituitary-adrenal axis, oxidative stress, reactive oxygen species, redox status, stress

## Abstract

Glucocorticoids released from the adrenal gland in response to stress-induced activation of the hypothalamic-pituitary-adrenal (HPA) axis induce activity in the cellular reduction-oxidation (redox) system. The redox system is a ubiquitous chemical mechanism allowing the transfer of electrons between donor/acceptors and target molecules during oxidative phosphorylation while simultaneously maintaining the overall cellular environment in a reduced state. The objective of this review is to present an overview of the current literature discussing the link between HPA axis-derived glucocorticoids and increased oxidative stress, particularly focussing on the redox changes observed in the hippocampus following glucocorticoid exposure.

## Introduction

The acute neuroendocrine response to adverse stress stimuli is characterized by the tripartite activation of the three stress axes including the autonomic sympathetic nervous system, the direct neural innervation of the adrenal cortex, and a cascade of hypothalamic hormonal messengers. Both the sympathetic system and the hypothalamic spinal adrenal axis utilize direct neural innervations of the adrenal medulla and cortex respectively to release adrenal catecholamines and prime the adrenal cortex for subsequent hormonal activation (Jansen et al., 1995; Buijs et al., 1999). This is initiated by neurosecretory neurons in the paraventricular nucleus of the hypothalamus, which release both corticotropin-releasing hormone and arginine vasopressin into the portal circulation of the pituitary gland. These two factors synergistically act on pituitary corticotroph cells to stimulate the release of the pro-opiomelanocortin peptide fragment, adrenocorticotropic hormone, into the circulation. Adrenocorticotropic hormone activates the melanocortin 2 receptor in the *zona fasciculata* of the adrenal cortex to initiate *de novo* synthesis and release of glucocorticoids, primarily cortisol in humans and corticosterone in rodents (Spiga et al., 2011). Together, this hormone cascade constitutes the hypothalamic-pituitary-adrenal (HPA) axis and is the primary system underlying stress physiology.

The physiological effects of corticosterone in the brain are canonically mediated through a near-ubiquitously expressed (with the exception of the suprachiasmatic nucleus of the hypothalamus) low affinity (KD ≈ 5.0 nM) glucocorticoid receptor (GR), and a regionally specific high affinity (KD ≈ 0.5 nM) mineralocorticoid receptor (MR) (Reul and de Kloet, 1985; Rose et al., 2012). Typically, these receptors reside in the cytoplasm heterocomplexed with heat shock proteins and immunophilins, which maintain the affinity of the hormone-binding domain (Pratt and Toft, 1997). The lipophilic steroid hormones are cell membrane permeable and bind these receptors, causing the dissociation of the chaperone proteins and translocation into the nucleus where the activated receptor complex forms GR and MR homo- or hetero-dimers that interact with specific glucocorticoid responsive elements in the promoter regions of genomic DNA. Both GR and MR elicit equivalent activity at glucocorticoid responsive elements and these interactions can result in transcriptional activation or repression of target genes depending on the cellular context (De Kloet et al., 1998). Transcriptional repression can also be mediated through protein-protein interactions specifically with activated GR and transcription factors such as NFκ B, offering a possible mechanism through which delineation of receptor function occurs between the GR and MR (van der Burg and van der Saag, 1996; De Kloet et al., 1998). Termination of the HPA response to stress is mediated through multiple negative feedback loops and utilizes both genomic and non-genomic actions of the GR (Calogero et al., 1988; Groeneweg et al., 2011). In circulation, adrenal glucocorticoids reach peak total plasma concentrations approximately 30 min after activation of the HPA axis (Qian et al., 2011). At the cellular level, these hormones act in conjunction with catecholamines to facilitate glucose availability and increase metabolic rate, which in turn increases spontaneous production of free radicals (Teague et al., 2007; Du et al., 2009).

## Free radical production

**Figure 1:**
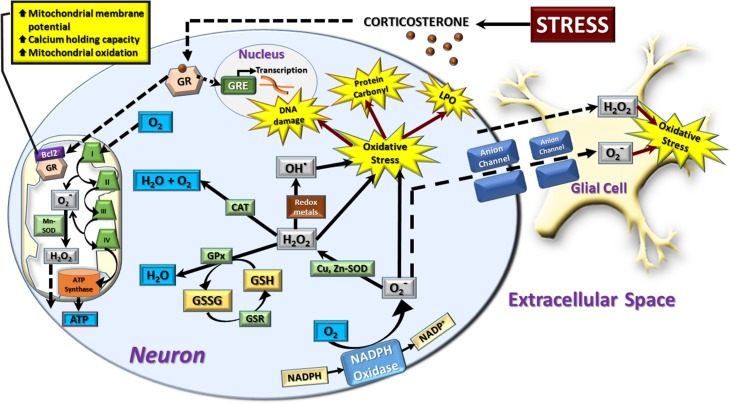
**Schematic representation of neural redox reactions**. Stress causes an increase in corticosterone which activates cytosolic glucocorticoid receptors (GR). These translocate into the nucleus to modulate gene transcription through glucocorticoid responsive elements (GRE), or co-localize with the anti-apoptotic Bcl-2 protein and translocate into the mitochondria. This increases mitochondrial membrane potential, calcium holding capacity, and mitochondrial oxidation. The increase in cellular metabolic rate promotes ATP synthesis in addition to spontaneous superoxide (O^−^_2_) production via complex I and III of the electron transport chain. This is dismutated to hydrogen peroxide (H_2_O_2_) by manganese superoxide dismutase (Mn-SOD) and can be further converted to hydroxyl radical (OH^−^) or reduced to water by the mitochondrial antioxidant pathway. In the cytosol, a major source of superoxide production is via the oxidation of NADPH via NADPH oxidase. Cytosolic superoxide is dismutated to hydrogen peroxide by copper, zinc-superoxide dismutase (Cu, Zn-SOD). Hydrogen peroxide is neutralized by catalase (CAT) or glutathione peroxidase (GPx) which oxidizes the reduced form of glutathione (GSH) to oxidized glutathione (GSSG). GSH is then regenerated from GSSG via the glutathione reductase (GSR) enzymatic system. Hydrogen peroxide can also interact with superoxide radicals and/or transition metals such as Fe^2+^ or Cu^2+^ to produce the highly toxic hydroxyl radical by Haber-Weiss and Fenton chemistry. An increase in the production of superoxide, hydrogen peroxide, and hydroxyl radicals leads to a state of cellular oxidative stress which causes oxidative damage to DNA, protein carbonyl formation, and membrane lipid peroxidation (LPO). Hydrogen peroxide is membrane permeable and moves freely from mitochondrial to cytosolic compartments, in addition to traversing the extracellular space to affect neighboring neurons and glial cells. Superoxide radicals can also induce oxidative stress in neighboring cells by diffusing through membrane-bound anion channels. The majority of neuronal and astrocytic reactive oxygen species are produced by mitochondrial oxidation, while other cell types such as microglia rely heavily on the cytosolic NADPH-oxidase system to produce a respiratory burst in response to invading pathogens. However, in comparison to glial cells, neurons display a relatively poor expression of endogenous antioxidants, making them more vulnerable to oxidative stress.

The process of aerobic metabolism utilizes oxygen to generate ATP in the mitochondrial electron transport chain (Halliwell and Gutteridge, 1989). During this process, 1–3% of all electrons “leak” from the electron transport chain to react with oxygen, generating superoxide radicals instead of being reduced to water (Liu et al., 2002; Muller et al., 2004; Cash et al., 2007). Although this occurs at both complex I and complex III of the electron transfer chain, the majority occurs at complex I where it is facilitated by succinate (Liu et al., 2002) (Figure 1). Most of the cellular superoxide is produced inside the inner mitochondrial membrane where the mitochondrial concentration of superoxide can be between 5–10 times that of the cytosol or nucleus (Cadenas and Davies, 2000). The remainder of mitochondrial superoxide is primarily formed by complex III on both sides of the mitochondrial membrane and by extra-mitochondrial flavoenzymes (Zimmerman and Granger, 1994; Cadenas and Sies, 1998; Brand et al., 2004). Superoxide then undergoes spontaneous or enzymatic dismutation via superoxide dismutase (SOD) to generate hydrogen peroxide. Although hydrogen peroxide is relatively stable, subsequent interactions with superoxide radicals and/or transition metals such as Fe^2+^ or Cu^2+^ induce production of the highly toxic hydroxyl radical by Haber-Weiss and Fenton chemistry. This radical has been suggested to cause more damage to biological systems than any other reactive oxygen species (ROS) due to the extreme reactivity and very short *in vivo* half-life of ≈9–10 ms (Pastor et al., 2000).

Outside the mitochondrion, there are three major processes responsible for the production of free radicals, principally in the form of reactive oxygen and nitrogen species. The first process involves the production of hydrogen peroxide as a by-product of fatty acid catabolism by peroxisomes (Ames et al., 1993; Wanders and Waterham, 2006). Although technically not a free radical, hydrogen peroxide is still classed as a ROS for its role in Fenton and Haber-Weiss chemistry (Cimen, 2008). Within the peroxisome, the majority of hydrogen peroxide is neutralized via canonical catalase activity or peroxidation to another catalase substrate (Wanders and Waterham, 2006; Valko et al., 2007). However, under some conditions hydrogen peroxide can avoid degradation and escape the peroxisome, ultimately leading to cellular and nucleic acid damage (Kasai et al., 1989). The second process involves the reliance of the innate immune system on the ability of phagocytic cells such as neutrophils to engulf and digest foreign pathogens. Following the encapsulation of the foreign body into a phagosome, neutrophils increase their oxygen consumption specifically to supply the dormant NADPH-oxidase with molecular oxygen (Dahlgren and Karlsson, 1999). This enzyme catalyzes the oxidation of NADPH to form two superoxide radicals which, together with reactive metabolites of superoxide including hydrogen peroxide and hypochlorite, constitutes the respiratory burst responsible for killing the pathogen (Ames et al., 1993; Dahlgren and Karlsson, 1999; Stadtman et al., 2007; Valko et al., 2007). Hayashi et al. (2008) have also demonstrated that NADPH-oxidase derived ROS can also be produced via a non-genomic mechanism following aldosterone administration in rat cardiac myocytes. The third process involves redox metals such as Fe^2/3+^, Cu^2+^, and Mn^2+^ which are essential for electron transfer in many enzymatic reactions, including the antioxidant enzymes of the oxidative cascade. However, these transitional metal ions can also undergo reactions resulting in the production of hydroxyl radicals (Rovira et al., 2007).

## The endogenous antioxidant system

In order to neutralize ROS, cells use a suite of enzymatic and non-enzymatic antioxidants, ultimately attempting to neutralize the radical by reduction to water. In the typical ROS reduction cascade, SOD is the top-tier antioxidant, catalyzing the dismutation of this radical to hydrogen peroxide. This is achieved through the transfer of electrons across the catalytic metal core of the enzymes to reduce the superoxide radicals. In mammals, the two main isoforms of SOD are the copper, zinc-SOD which are found throughout most cell compartments, and the manganese-SOD that is specific for mitochondria. Catalase enzymes are centered around an iron-containing ferriheme group that acts as the transition metal during the reduction of hydrogen peroxide. Access to this active site is fairly specific as the channel opening is narrow and does not allow the passage of large molecules. High concentrations of superoxide anions are able to inactivate catalase by oxidizing the heme group in the active site. To prevent this, catalase binds NADPH to maintain this group in the reduced state (Nordberg and Arner, 2001; Fridovich et al., 2007). Hydrogen peroxide can also be reduced directly by both peroxiredoxins, which allow the oxidation of an active cysteine thiol group to degrade one molecule of hydrogen peroxide into two molecules of water, and the glutathione-glutathione peroxidase system. Reduced glutathione (GSH) is the most abundant intracellular thiol-based antioxidant which protects cells against oxidative stress by acting as a substrate for the selenium-containing glutathione peroxidase, subsequently forming oxidized glutathione disulphide (GSSG). In turn, GSSG is regenerated to GSH by glutathione reductase in a NADPH-dependent mechanism (Barycki et al., 2007) (Figure 1). The cellular concentrations of this soluble tripeptide range from 1 to 11 mM in the cytosol, 3–15 mM in the nucleus, and 5–11 mM in the mitochondria, although mitochondrial GSH requires membrane transport even against a concentration gradient (Shen et al., 2005; Valko et al., 2007). Glutathione also acts as a substrate for the glutaredoxins, which reduce proteins that have been glutathionylated by reducing GSSG to a mixed disulphide protein and GSH. As the occurrence of mixed disulphides increases with increasing concentrations of GSSG, the ratio of the reduced to oxidized fractions (GSH/GSSG) of GSH within cells is often used as a reliable indicator of redox imbalance and has been shown to strongly influence cell cycle progression in proliferating cells (Menon et al., 2003; Öztürk and Gümüslü, 2004; Rose et al., 2012). The transcription factor nuclear factor-erythroid-2-related factor 2 (Nrf2) is essential for the coordinated induction of cytoprotective enzymes and related proteins in response to oxidative and electrophilic stresses (Itoh et al., 1999; Uruno and Motohashi, 2011). This transcription factor regulates a battery of redox genes such as the glutathione synthesis enzyme gamma-glutamylcysteine synthetase, glutathione peroxidase, glutathione disulphide reductase, glutathione *S*-transferase, thioredoxin-1, and heme oxygenase-1 through their antioxidant response element (Rushmore et al., 1990; Inamdar et al., 1996; Moinova and Mulcahy, 1999; Kim et al., 2003; Kwak et al., 2003). Under basal conditions, Nrf2 activity is sequestered in part by the actin-associated Keap1 protein within the cytoplasm. Activation of Nrf2 in response to oxidative and electrophilic agents is thought to be initiated by disruption of this Nrf2-Keap1 complex, releasing Nrf2, which translocates into the nucleus to regulate the expression of downstream targets.

## Physiological role of reactive oxygen species

Although there is a general negative connotation associated with ROS production, they have important cellular functions under normal physiological conditions. Even low levels of the extremely reactive hydroxyl radical have been shown to activate guanylate cyclase, stimulating the production of a cGMP second messenger cascade (Mittal and Murad, 1977). In fact, the physiological roles of ROS vary significantly, ranging from specific oxidations of cysteine groups affecting enzyme activity and function, to cellular redox sensing in the determination of cell differentiation fate (Nicotera et al., 1985; Dalton et al., 1999; Wang et al., 2011). Progression of the cell cycle itself has demonstrated dependence on radicals produced by NADPH-oxidase modulating mitogenic pathways (Burhans and Heintz, 2009). Several transcription factors are also regulated directly by ROS-induced modifications, thereby modulating the downstream expression of several gene families (Dalton et al., 1999). Notably, the dimerized protein products of immediate-early response genes FOS and JUN, AP-1, is activated by ROS through redox reactions and post-translational modification of the individual FOS and JUN proteins (Buscher et al., 1988; Abate et al., 1991; Devary et al., 1991). Under normal conditions, any excessive ROS not participating in these physiological functions are reduced by the antioxidant system. However, an imbalance between the production of ROS and the ability of the antioxidant defense system to readily detoxify the reactive intermediates, termed oxidative stress, leads to damage of biological macromolecules and dysregulation of normal metabolism (Sies, 1997; Nordberg and Arner, 2001).

## Adrenal glucocorticoids and oxidative stress

Increased secretion of adrenal glucocorticoids following physical and/or psychological stress exposure subsequently liberates glucose through gluconeogenesis, glycogenolysis, and lipolysis (Teague et al., 2007). Although increased metabolism alone generates ROS, glucocorticoids have demonstrated both direct and indirect modulatory roles in the onset of oxidative stress. Furthermore, both chronic oxidative stress and glucocorticoid exposure promote gliogenesis over neurogenesis in hippocampal neural stem cell progenitors and may be the direct result of accumulated mitochondrial oxidative stress (Wang et al., 2011; Chetty et al., 2014). Glial cells play an important but poorly understood role in the modulation of neuronal redox state. It has recently been shown that astrocyte-derived L-lactate potentiates NMDA receptor activity by modulating neuronal redox status (Yang et al., 2014). This neuron-glia interaction can also increase noradrenaline release from the locus ceruleus and hypothalamic ATP production (Cortes-Campos et al., 2011; Tang et al., 2014). Furthermore, Reyes et al. (2012) have demonstrated that neuronal NADPH oxidase-derived superoxide can traverse the extracellular space to modulate the redox state in neighboring neurons and astrocytes (Figure 1). However, in comparison to astrocytes, neurons are known to have relatively poor expression of endogenous antioxidants, making them highly susceptible to oxidative stress.

Glucocorticoids induce neuronal oxidative stress directly through enhanced mitochondrial respiration and oxidative phosphorylation. This was demonstrated clearly in a study by Du et al. (2009), showing that acute incubation of cortical neurons with corticosterone increased mitochondrial oxidation, membrane potential, and calcium-holding capacity in a dose and time-dependent manner (Figure 1). This was further clarified by You et al. (2009) using the oxidation product dichlorofluorescien as a ROS indicator in organotypic hippocampal slice cultures exposed to the synthetic glucocorticoid, dexamethasone, and the glucocorticoid receptor antagonist, RU486. The single and combination use of these compounds demonstrated that hippocampal neuronal death, marked by propidium iodide, was selectively induced by glucocorticoid exposure, while other steroid hormones had no effect. Acute incubation with dexamethasone increased the hippocampal oxidative status by approximately 200% in a dose dependent manner, an effect that was ameliorated with pre-treatment of RU486 or the ROS-scavenger, N-acetyl-L-cysteine. Furthermore, 4 h of dexamethasone incubation, induced the highest increase in oxidative status, with concurrent gene expression up-regulation of the ROS-producing enzyme NADPH-oxidase, while the antioxidant enzyme glutathione peroxidase was significantly down-regulated (You et al., 2009). This indicates that the increase in oxidative status is produced by a glucocorticoid-dependent and transcriptional increase in pro-oxidative drive, with concurrent inhibition of the antioxidant defense system, ultimately leading to increased neuronal cell death. Cortical and hippocampal neural cultures have also established that 24 h of glucocorticoid exposure increases basal ROS production and exacerbates the concomitant ROS produced by adriamycin redox cycling, which negatively affects survival in hippocampal neurons (McIntosh and Sapolsky, 1996). This is supported by *in vivo* evidence that administration of exogenous corticosterone over a 14 day period increases hippocampal oxidative indicators including lipid peroxidation and protein carbonyls, while the enzymatic antioxidants SOD, catalase, and glutathione peroxidase activities are all decreased (Sato et al., 2010). This study, performed by Sato et al. (2010), also demonstrated increased apoptosis and decreased glucocorticoid receptor expression, a hallmark of chronically high glucocorticoids, in the hippocampus. Interestingly, this study also utilized a serum measurement of iron-induced superoxide formation in blood serum, establishing that this peripheral marker increased with chronic corticosterone administration. Further *in vivo* studies have shown that both corticosterone administration and restraint stress for 21 days induce an overall decrease in GSH in addition to SOD, catalase, glutathione transferase, and glutathione reductase activities in whole brain, liver, and heart tissues (Zafir and Banu, 2009). This study, by Zafir and Banu (2009), also demonstrated that these treatments increased lipid peroxidation and oxidized protein carbonyl groups in the same tissues. However, in the brain, this overall oxidative increase is likely due to increases in specific subregions. For example, Mendez-Cuesta et al. (2011) used an acute immobilization stress to induce increased lipid peroxidation and decreased SOD activity in a highly oxidative-vulnerable region, the striatum. Interestingly, they found that the decrease in SOD activity was exclusively due to the mitochondrial isoform, manganese-SOD, while the copper, zinc-SOD showed little change. Furthermore, Lucca et al. (2009) used a chronic mild stress regime to show that SOD decreased and oxidized protein carbonyl groups increased in several cerebral regions including the striatum and hippocampus, while the cerebellum remained largely unaffected. We have also observed the induction of regional specific oxidative stress in the hippocampus but not the amygdala following an acute restraint stress, demonstrating that this process is not exclusive to chronic stress exposure (Spiers et al., 2013). Recently, exposure to glucocorticoids or dexamethasone demonstrated inhibitory action over the Nrf2-dependent antioxidant pathway, causing an increase in hepatic and osteoblastic cell ROS, an effect attenuated by exogenous sulforaphane (Kratschmar et al., 2012; Lin et al., 2014).

Interestingly, ROS attenuate the glucocorticoid-induced down-regulation of pro-opiomelanocortin in pituitary corticotrophs, thereby promoting an increase in HPA axis activity via reduced negative feedback (Asaba et al., 2004). The expression and nuclear internalization of GR have also demonstrated susceptibility to highly pro-oxidative environments (Okamoto et al., 1999; Zhou et al., 2011). Using a cultured fluorescently labeled chimeric GR, Okamoto et al. (1999) demonstrated that nuclear translocation of GR following acute dexamethasone treatment is impaired in the presence of hydrogen peroxide. This effect was reduced in the presence of exogenous antioxidants or following substitution of serine for a redox-sensitive cysteine residue. The dissociation of heat shock proteins from the cytosolic GR was also impaired in a pro-oxidative environment, indicating that there may be multiple redox regulatory roles involved in the cellular response to glucocorticoids (Okamoto et al., 1999 reviewed in Tanaka et al., 1999). These observations highlight that maintenance of a balanced redox state is critical for normal cellular homeostatic function within the neuroendocrine system.

## Conclusions

Although oxidative stress and elevated glucocorticoids are both observed in a number of chronic pathologies, the delineation between physiological function and pathological insult is complex and remains unclear. In particular, the role of ROS in neuron-neuron and neuron-glia interactions is an area that requires attention. Based on the observations of increased gliogenesis in the presence of high levels of glucocorticoids, the mobility of ROS in the extracellular space, and the relative paucity of neuronal endogenous antioxidants, we suggest that glia may be playing an active role in response to neuronal oxidative stress. This may have important implications for neurodegenerative conditions involving redox-sensitive regions such as the hippocampus. These observations highlight that maintenance of a balanced redox state is critical for normal cellular homeostatic function and relies heavily on hormonal cues from the neuroendocrine stress system.

## Author contributions

Author Jereme G. Spiers managed the literature searches and wrote the first draft of the manuscript. Author Hsiao-Jou Cortina Chen produced the graphic and revised the manuscript. Author Conrad Sernia and Nickolas A. Lavidis critically revised the manuscript. All authors have approved the final version of the manuscript for journal submission.

### Conflict of interest statement

The authors declare that the research was conducted in the absence of any commercial or financial relationships that could be construed as a potential conflict of interest.
